# TGF-β signalling and PEG10 are mutually exclusive and inhibitory in chondrosarcoma cells

**DOI:** 10.1038/s41598-017-13994-w

**Published:** 2017-10-18

**Authors:** Naohiro Shinohara, Shingo Maeda, Yuhei Yahiro, Daisuke Sakuma, Kanehiro Matsuyama, Katsuyuki Imamura, Ichiro Kawamura, Takao Setoguchi, Yasuhiro Ishidou, Satoshi Nagano, Setsuro Komiya

**Affiliations:** 1 Department of Medical Joint Materials, Kagoshima University, Kagoshima, Japan; 2 Department of Orthopaedic Surgery, Kagoshima University, Kagoshima, Japan; 3 The Near-Future Locomotor Organ Medicine Creation Course, Kagoshima University, Kagoshima, Japan

## Abstract

Histological distinction between enchondroma and chondrosarcoma is difficult because of a lack of definitive biomarkers. Here, we found highly active transforming growth factor-β (TGF-β) and bone morphogenetic protein (BMP) signalling in human chondrosarcomas compared with enchondromas by immunohistochemistry of phosphorylated SMAD3 and SMAD1/5. In contrast, the chondrogenic master regulator SOX9 was dramatically down-regulated in grade 1 chondrosarcoma. Paternally expressed gene 10 (*PEG10*) was identified by microarray analysis as a gene overexpressed in chondrosarcoma SW1353 and Hs 819.T cells compared with C28/I2 normal chondrocytes, while TGF-β1 treatment, mimicking higher grade tumour conditions, suppressed *PEG10* expression. Enchondroma samples exhibited stronger expression of *PEG10* compared with chondrosarcomas, suggesting a negative association of *PEG10* with malignant cartilage tumours. In chondrosarcoma cell lines, application of the TGF-β signalling inhibitor, SB431542, increased the protein level of PEG10. Reporter assays revealed that PEG10 repressed TGF-β and BMP signalling, which are both SMAD pathways, whereas PEG10 knockdown increased the level of phosphorylated SMAD3 and SMAD1/5/9. Our results indicate that mutually exclusive expression of PEG10 and phosphorylated SMADs in combination with differentially expressed SOX9 is an index to distinguish between enchondroma and chondrosarcoma, while PEG10 and TGF-β signalling are mutually inhibitory in chondrosarcoma cells.

## Introduction

Chondrosarcoma is the second most common primary malignant bone tumour that is characterised by formation of cartilaginous extracellular matrix (ECM). It represents 10–20% of malignant bone lesions with an incidence of 1 in 200,000 people per year^1,2^. Chondrosarcoma is classified into three histological grades based on cellularity, nuclear atypia, and pleomorphism. Grade 1 chondrosarcomas grow slowly and rarely metastasise, whereas grade 2 or 3 tumours develop more aggressively and are associated with high rates of metastasis^3,4^. Because of the abundant ECM, low rate of cell proliferation, and poor vascularity, chondrosarcomas seldom respond to chemotherapy or radiotherapy^5–7^. Therefore, wide surgical resection remains the only curative treatment for patients with these tumours^8^. However, even after adequate surgery, the prognosis of chondrosarcomas depends on the tumour grade. Ten-year survival for patients with grade 1 is excellent, but it is only 64% for grade 2 chondrosarcomas and 29% for grade 3^9^. These poor prognoses can in part be explained by the high frequency of metastasis in high-grade tumours. Enchondroma, a benign counterpart of chondrosarcoma, is a cartilage neoplasm that can develop in any bone formed by endochondral ossification. It is commonly found in around 3% of routine knee magnetic resonance imaging examinations^10,11^. Because the majority of orthopaedic oncologists follow asymptomatic enchondromas by serial imaging alone to rule out progression^12^, it is clinically crucial to distinguish low-grade chondrosarcoma from enchondroma. However, histological distinction is often difficult and sometimes even impossible for skilled pathologists because these tumours harbour similarities in cellularity, cytology, and cartilaginous ECM^13–18^. Moreover, in some borderline cases, it is difficult to distinguish low-grade from high-grade chondrosarcomas because the grading criteria are not necessarily definitive^16^. Because it is a recent trend that grade 1 chondrosarcomas can be treated by curettage instead of wide resection, followed by adjuvant local cryosurgery or phenolisation^19,20^, it is also crucial to distinguish grade 1 from grade 2 chondrosarcomas.

To overcome such a frequent diagnostic dilemma of orthopaedic oncologists, researchers have made efforts to identify specific molecular markers to distinguish and diagnose the grades of chondrosarcomas by immunohistochemistry (IHC). We previously reported that enchondromas express GADD45β, and that its level decreases in chondrosarcoma according to the malignancy grade^14^. Other groups have reported differential expression of the following molecules between enchondromas and grade 1 chondrosarcomas: periostin^21^, Runx2, Indian Hedgehog^22^, C-propeptides of procollagen Iα1 and IIα1^23^, MCM6^24^, PTHrP, Bcl-2^25^, CD44s^26^, and components of the transforming growth factor-β (TGF-β) pathway^27,28^. However, no definitive biomarkers have been established so far.

Members of the TGF-β family, including bone morphogenetic proteins (BMPs), transduce signals through type II and type I receptors to activate receptor-regulated Smads (R-Smads) by phosphorylation. TGF-βs activate Smad2/3, and Smad1/5/9 are the downstream mediators of BMP signalling. Activated R-Smads translocate into the nucleus after formation of a trimeric complex with a common Smad4 (Co-Smad) to regulate the transcription of target genes^29^. Loss-of-function of BMP signalling in mouse cartilage impairs chondrogenesis^30–32^, and the TGF-β pathway promotes chondrogenesis by enabling Smad3 to form an active transcriptional complex with CBP/p300 and the chondrogenic master regulator, Sox9^33^. In human chondrosarcoma, TGF-β and BMP pathways are active^27,28^. In general, the degree of tumour malignancy negatively correlates with the level of cellular differentiation; therefore, we hypothesised that the chondrogenic property of TGF-β family members might have crucial roles in determining the differentiation and malignancy status of chondrogenic tumours. However, importantly, the potential difference in expression levels of TGF-β family members among enchondromas and grade 1 chondrosarcomas is not well studied. In addition, little information is available concerning downstream target genes of the TGF-β family, which mediate the malignant phenotypes of cartilage tumours.

The aim of this study was to identify downstream molecules of TGF-β and/or BMP signalling pathways that are differentially expressed between enchondroma and grade 1 chondrosarcoma. Here, we found that paternally expressed gene 10 (*PEG10*) was strongly expressed in human enchondromas, but it was significantly diminished in grade 1 chondrosarcomas. *In vitro*, PEG10 expression was suppressed by TGF-β1 stimulation, whereas PEG10 inhibited the canonical SMAD pathway of TGF-β and BMP signalling. Our results showed mutually exclusive expression patterns and inhibitory roles of TGF-β and PEG10 in chondrosarcomas.

## Results

### Status of chondrocytic differentiation and activity of TGF-β/BMP signalling in enchondroma and chondrosarcoma

**Figure 1:**
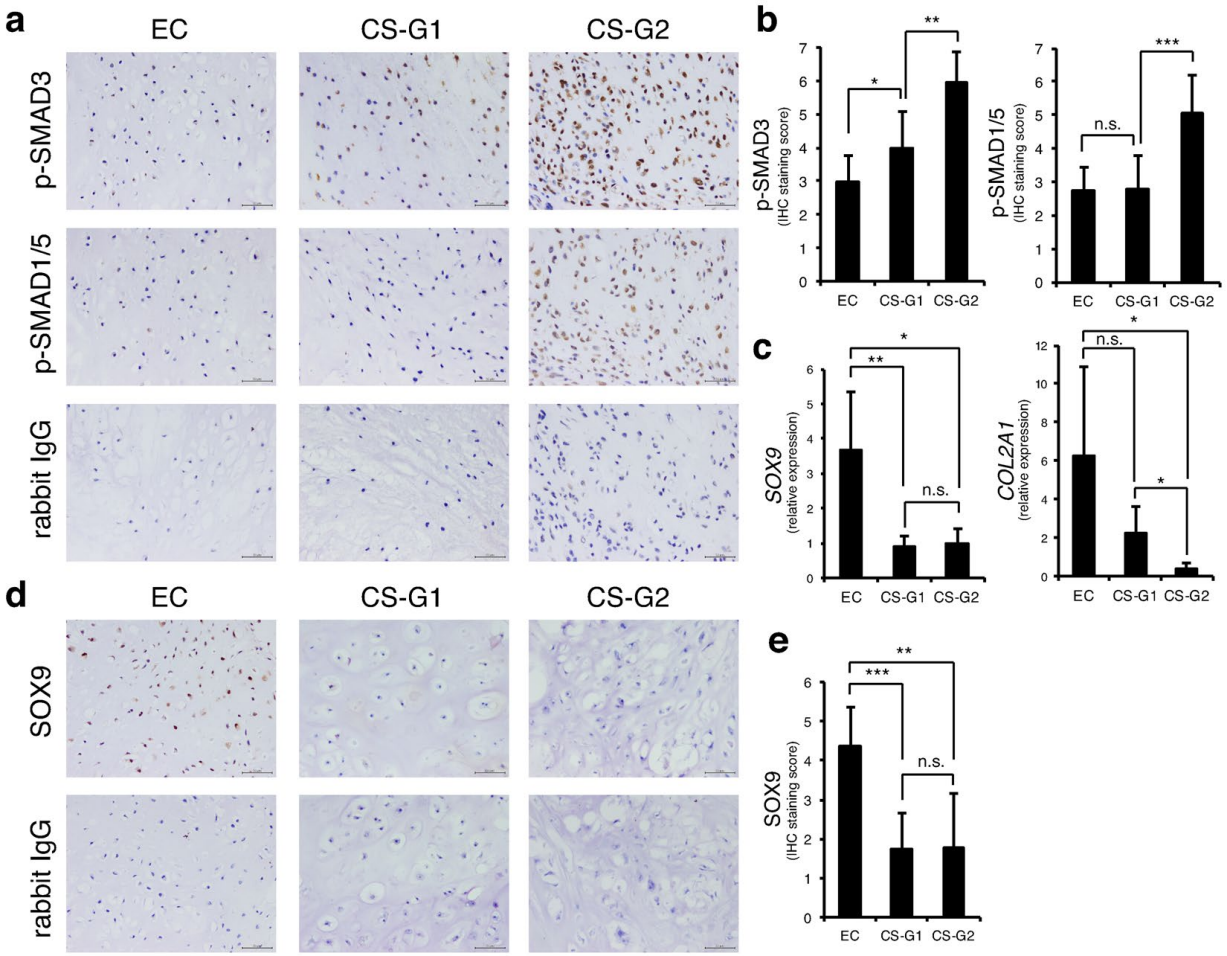
SMAD3 and SMAD1/5 are strongly phosphorylated in chondrosarcoma. (**a**) Immunohistochemistry (IHC) of phosphorylated (p-) SMAD3 and p-SMAD1/5 in enchondroma and chondrosarcoma specimens. Rabbit IgG was used as a negative control. EC, enchondromas (n = 7); CS-G1, grade 1 chondrosarcomas (n = 11); CS-G2, grade 2 chondrosarcomas (n = 7). Scale bar = 50 μm. (**b**) Summed scores of the percentage and intensity of positive staining in IHC. (**c**) Expression of *SOX9* and *COL2A1* was examined by reverse transcription-quantitative polymerase chain reaction (RT-qPCR). EC (n = 6); CS-G1 (n = 5); CS-G2 (n = 6). (**d**) IHC of SOX9 in enchondroma and chondrosarcoma specimens. Rabbit IgG was used as a negative control. EC, enchondromas (n = 7); CS-G1, grade 1 chondrosarcomas (n = 11); CS-G2, grade 2 chondrosarcomas (n = 7). Scale bar = 50 μm. (**e**) Summed scores of the percentage and intensity of positive staining in IHC. *P < 0.05; **P < 0.01; ***P < 0.001; n.s., not significant.

The activity of R-SMADs in TGF-β and BMP signalling is higher in high-grade chondrosarcomas than in low grades^28^. To confirm this trend in our chondrosarcoma samples and to investigate the possible difference between benign enchondroma and malignant chondrosarcoma, we evaluated the rate of phosphorylated (activated) SMAD3 (p-SMAD3) and SMAD1/5 (p-SMAD1/5) in formalin-fixed, paraffin-embedded (FFPE) specimens by IHC. Staining of p-SMAD3 was weakly detected in enchondroma (score 3), but it was measurably increased in grade 1 chondrosarcoma (score 4) and further augmented in grade 2 chondrosarcoma at score 6 (Fig. 1a,b). Expression of p-SMAD1/5 showed no statistical difference between enchondroma and grade 1 chondrosarcoma. However, it was almost doubled in grade 2 chondrosarcoma (Fig. 1a,b). Because both R-SMADs in TGF-β and BMP signalling systems are chondrogenic drivers, we examined expression of chondrogenic marker genes by quantitative polymerase chain reaction (qPCR). The chondrogenic master regulator gene *SOX9* was strongly expressed in enchondromas, but it was significantly diminished in grade 1 chondrosarcomas and not associated with the grade (1 or 2) (Fig. 1c). The cartilage-specific collagen gene, *COL2A1*, was also appreciably expressed in enchondromas, and its expression tended to decrease in grade 1 chondrosarcoma, but not significantly, whereas it was dramatically attenuated in grade 2 chondrosarcomas (Fig. 1c). The differential expression pattern of SOX9 was confirmed at the protein level by IHC, that its strong expression was detected in enchondromas whereas it was diminished in chondrosarcomas (Fig. 1d,e). These results showed that chondrosarcomas lose their chondrogenic property as they gain the malignant phenotype, suggesting that chondrosarcoma does not respond to TGF-β/BMP signalling for chondrogenesis. To determine which components of the TGF-β/BMP signalling pathway were responsible for the high activity of SMADs in chondrosarcomas, we examined the expression profiles of TGF-β ligands, BMP ligands, TGF-β type I receptors, BMP type I receptors, TGF-β-regulated R-SMADs, SMAD4, and BMP-regulated R-SMADs (Supplementary Fig. S1a–g). However, none of the analysed genes showed increased expression in chondrosarcomas compared with enchondromas, raising the possibility that molecules that inhibit phosphorylation of SMADs were decreased to accelerate SMADs activation.

### Screening for genes that are abundantly expressed in chondrosarcoma cells and that are suppressed by TGF-β stimulation

We hypothesised that inhibitory molecules in TGF-β signalling were decreased by the increased TGF-β signalling in chondrosarcomas compared with enchondromas in a negative feedback fashion. Because an enchondroma cell line has not been established, we employed two chondrosarcoma cell lines, SW1353 and Hs 819.T. If our hypothesis is true, the inhibitory molecules should be expressed in these cell lines at a substantial level and be down-regulated by the addition of exogenous TGF-β ligand mimicking a higher grade of chondrosarcoma. In advance of the target gene screening, we characterised SW1353 and Hs 819.T cells for their differentiation status and TGF-β responsiveness. In a chondrogenic micromass three-dimensional (3-D) culture system, the normal chondrocyte line C28/I2 formed a cartilage matrix positive for alcian blue staining, whereas chondrosarcoma cells failed to maintain the micromass structure (Supplementary Fig. S2a, left panel). These cells were treated with TGF-β1 for 48 h followed by microarray analysis. Microarray analysis showed that, although the mRNA level of *COL2A1* was comparable among the tested cells, C28/I2 cells expressed a high level of *HAS2*, which encodes hyaluronan synthase, and which was further increased by TGF-β1, whereas both SW1353 and Hs 819.T cells showed only weak expression (Supplementary Fig. S2b), reflecting the result of alcian blue staining. Instead, chondrosarcoma cell lines were positive for late chondrocyte markers such as alkaline phosphatase (ALP) activity or *COL10A1* expression, both of which were strongly increased by TGF-β1 treatment, indicating activation of an abnormal differentiation program in chondrosarcoma cells (Supplementary Fig. S2b). Because TGF-β1 stimulation did not induce normal chondrogenic differentiation of SW1353 and Hs 819.T cells, we checked the responsiveness against TGF-β signalling by examining a representative canonical SMAD axis target gene, *SERPINE1* (PAI1), to examine whether all cell lines responded sufficiently to TGF-β1 ligand stimulation. TGF-β1 treatment increased expression of *SERPINE1* in all tested cells, while chondrosarcoma cell lines showed a significantly higher basal expression level, suggesting that the canonical TGF-β pathway was more active in chondrosarcoma cell lines (Supplementary Fig. S2b).

In this model, we identified 17 genes out of 48,122 (Table 1), whose expression patterns met the criteria. Among these genes, we focused on *PEG10* because of the following reasons. First, the *Peg10* gene is strongly expressed in cartilage primordium of mouse embryos^34^. Second, accumulating evidence suggests that PEG10 plays an important role in the promotion of tumour growth in various cancers including hepatocellular carcinoma, lung cancer, and prostate cancer^35–37^. Finally, PEG10 interacts with TGF-β type I and II receptors, and interferes with their signalling^38^. This evidence led us to investigate the possible association and roles of PEG10 in chondrosarcoma.

**Table 1:** Results of microarray analysis: genes upregulated in chondrosarcoma cells and downregulated by TGF-β1 treatment.

Gene symbol	Gene description	probe signal intensity				
		C28/I2 + mock	SW1353 + mock	SW1353 + TGF-β1	Hs 819.T + mock	Hs 819.T + TGF-β1
*CEACAMP6*	carcinoembryonic antigen-related cell adhesion molecule pseudogene 6	11.644251	849.7766	293.17218	805.261	188.41696
*CHI3L1*	chitinase 3-like 1 (cartilage glycoprotein-39)	30.32458	891.2907	193.51378	733.03796	274.57275
*DUSP10*	dual specificity phosphatase 10	264.37048	1730.9897	749.4703	1657.7657	691.1046
*F2RL2*	coagulation factor II (thrombin) receptor-like 2	51.60757	1596.104	743.29755	2073.8364	792.52045
*GPNMB*	glycoprotein (transmembrane) nmb	69.241974	456.36475	134.65535	437.88937	143.89958
*IL36B*	interleukin 36, beta	14.552366	708.716	261.0168	402.83282	134.94543
*MAD1L1*	MAD1 mitotic arrest deficient-like 1 (yeast)	170.33104	1065.3365	415.22104	1033.9989	438.05844
*MIR221*	microRNA 221	91.36347	869.62396	263.8181	1107.2083	283.76727
*MME*	membrane metallo-endopeptidase	30.921976	613.5276	265.4864	757.2124	338.64307
*MMP3*	matrix metallopeptidase 3 (stromelysin 1, progelatinase)	20.330805	878.2055	197.87737	1292.2312	266.2604
*MOSPD1*	motile sperm domain containing 1	196.03433	1332.8429	626.03235	1398.9138	680.8544
*PDE5A*	phosphodiesterase 5 A, cGMP-specific	14.682203	415.84045	98.5719	706.4834	166.55884
*PEG10*	paternally expressed gene 10	92.65147	460.203	206.1215	459.444	197.3221
*PHLDA1*	pleckstrin homology-like domain, family A, member 1	103.9472	489.51953	215.93834	433.7974	207.7058
*PSG4*	pregnancy specific beta-1-glycoprotein 4	28.558039	1003.54065	367.46582	1020.25616	259.7936
*RAB27B*	ras-related protein Rab-27B	57.8036	1387.0364	239.84311	1366.6107	252.79196
*VAT1L*	vesicle amine transport protein 1 homolog (T. californica)-like	165.3608	801.96643	307.95685	824.4419	367.76886

C28/I2 cells were mock treated, while SW1353 and Hs 819.T cells were treated with or without TGF-β1 (1 ng/ml) for 48 h, followed by mRNA purification and microarray analysis. Genes were identified based on a 4-fold higher probe signal in both SW1353 and Hs 819.T cells compared with C28/I2 cells with notable intensity of >400, which decreased to less than 0.5-fold upon TGF-β1 induction.

### *PEG10* is highly expressed in enchondroma and decreased in chondrosarcoma

**Figure 2:**
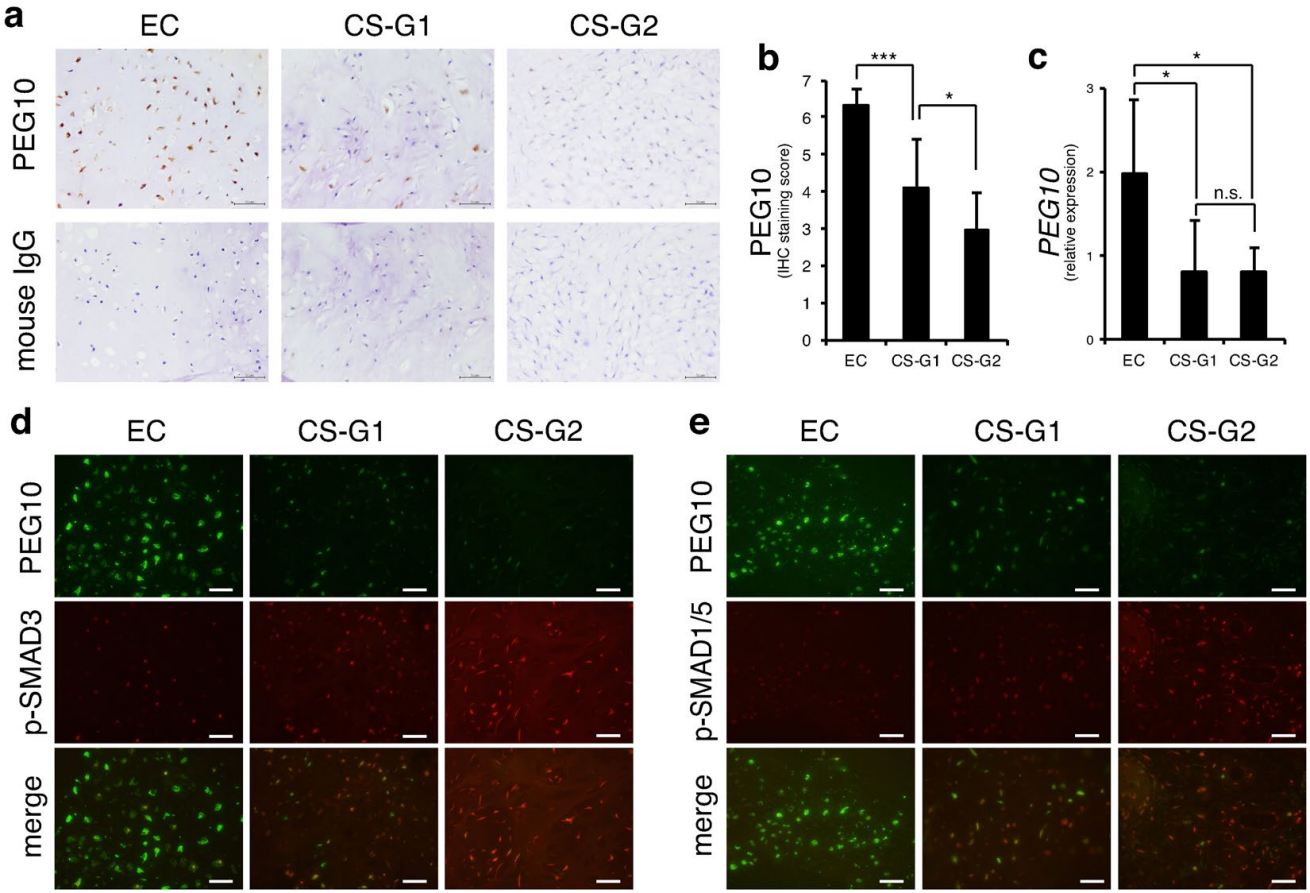
PEG10 accumulates in enchondromas but is eliminated in chondrosarcomas. (**a**) IHC of PEG10 in enchondroma and chondrosarcoma specimens. Normal mouse IgG was used as a negative control. EC, enchondromas (n = 7); CS-G1, grade 1 chondrosarcomas (n = 11); CS-G2, grade 2 chondrosarcomas (n = 7). Scale bar = 50 μm. (**b**) Summed score of the percentage and intensity of positive staining in IHC. (**c**) Expression of *PEG10* was examined by RT-qPCR. EC (n = 6); CS-G1 (n = 5); CS-G2 (n = 6). *P < 0.05; ***P < 0.001; n.s., not significant. (**d**,**e**) Double immunofluorescence (IF) of PEG10 and p-SMAD3 or p-SMAD1/5 in enchondroma and chondrosarcoma specimens. Signals of PEG10 were detected by an anti-mouse Alexa Fluor 488 antibody (green), while p-SMADs were stained by an anti-rabbit Alexa Fluor 568 antibody (red). Scale bar = 25 μm.

We examined the protein levels of PEG10 in clinical tissue samples of enchondroma and chondrosarcoma by IHC. PEG10 was strongly positive in enchondromas with staining scores of >6 (Fig. 2a,b). In grade 1 chondrosarcomas, expression of PEG10 was moderately but significantly decreased (score 4) and decreased further in grade 2 chondrosarcomas (score 3) (Fig. 2a,b). This trend was essentially reproduced in the mRNA levels of frozen specimens, although there was no significant difference between grades 1 and 2 (Fig. 2c). To confirm the mutually exclusive expression pattern of PEG10 and p-SMADs in tumour specimens, we performed double immunofluorescence (IF) of PEG10 and p-SMADs. As expected, only a small population of tumour cells showed co-expression of PEG10 and p-SMAD3 (Fig. 2d) or PEG10 and pSMAD1/5 (Fig. 2e). PEG10 was dominantly expressed in enchondromas, whereas p-SMADs were detected in chondrosarcomas. Importantly, the staining pattern of double IF was clearly different between enchondromas and grade 1 chondrosarcomas. These results of IHC and IF staining for p-SMAD3, p-SMAD1/5, and PEG10 showed mutually exclusive expression patterns that distinguish between enchondromas and grade 1 chondrosarcomas.

### PEG10 is expressed in chondrosarcoma cells and is diminished by TGF-β treatment

**Figure 3:**
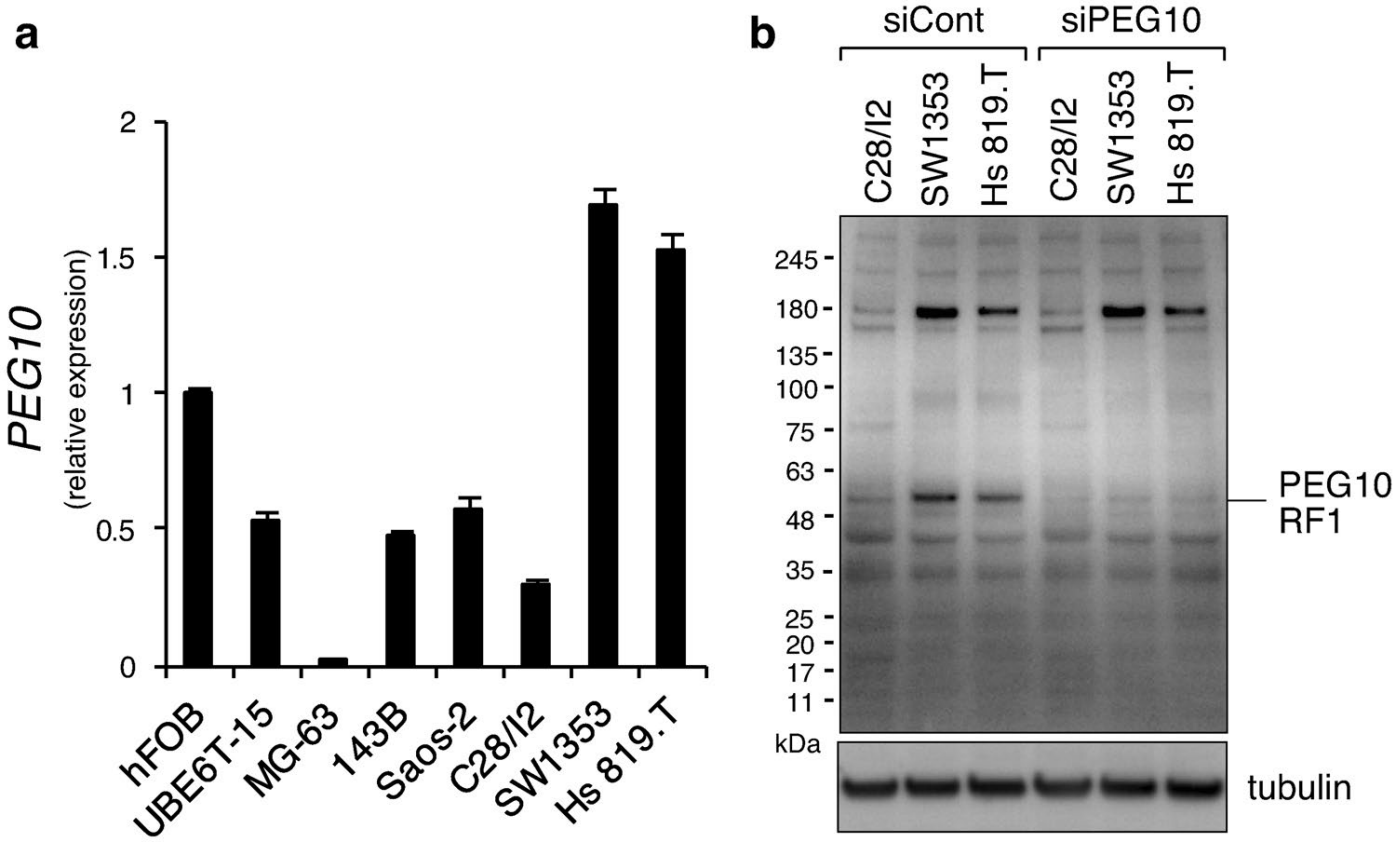
PEG10 is overexpressed in chondrosarcoma cell lines SW1353 and Hs 819.T. (**a**) Expression of *PEG10* in the indicated cell lines was examined by RT-qPCR (n = 3). (**b**) A mixture of four independent siRNAs against *PEG10* was transfected into the indicated cells, followed by immunoblotting for PEG10. A specific band for PEG10-RF1, which was diminished by knockdown, is indicated. Tubulin served as a loading control. The blot for tubulin was cropped and the full-length blot is presented in Supplementary Figure S5.

**Figure 4:**
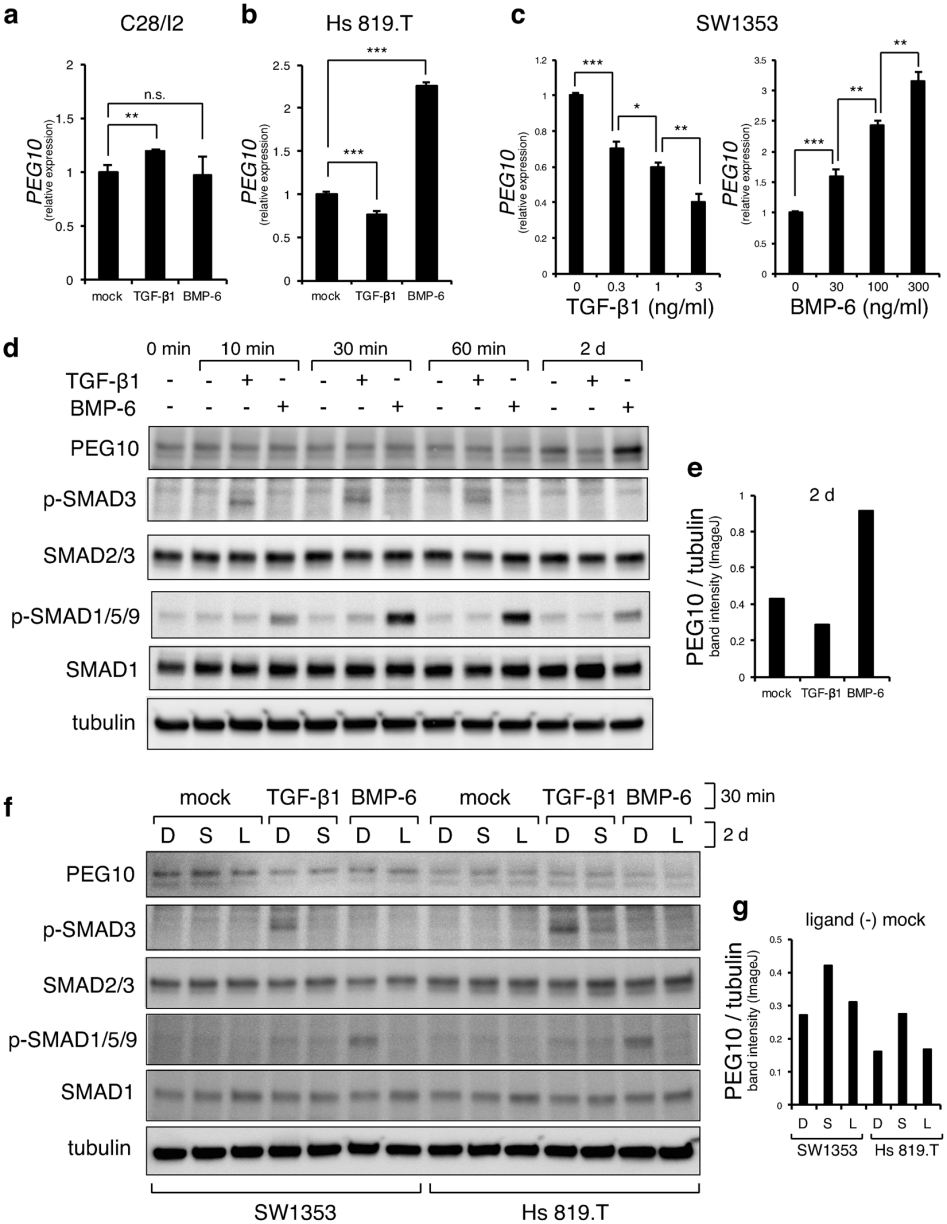
PEG10 is negatively regulated by TGF-β signalling. (**a**) Expression of *PEG10* in C28/I2 cells was examined by RT-qPCR at 2 days after application of TGF-β1 (1 ng/ml) or BMP-6 (100 ng/ml) (n = 3). (**b**) Expression of *PEG10* in Hs 819.T cells was examined by RT-qPCR at 7 days after application of TGF-β1 (1 ng/ml) or BMP-6 (100 ng/ml) (n = 3). (**c**) Expression of *PEG10* in SW1353 cells was examined by RT-qPCR at 2 days after application of TGF-β1 or BMP-6 at the indicated concentrations (n = 3). *P < 0.05; **P < 0.01; ***P < 0.001 (**c**,**d**); n.s., not significant. (**d**) Expression of PEG10 protein in SW1353 cells was examined by immunoblotting at the indicated time points after application of TGF-β1 (1 ng/ml) or BMP-6 (100 ng/ml). Blots were cropped and the full-length blots are presented in Supplementary Figure S5. Band intensities of PEG10 and tubulin at 2 days (lanes 11–13) were quantified using ImageJ software (**e**). The intensity of PEG10 was normalised to that of tubulin. (**f**,**g**) Chondrosarcoma cell lines were treated with DMSO (D, 0.01%), SB431542 (S, 1 μM), or LDN193189 (L, 0.1 μM) in serum-free medium containing ITS supplement overnight, followed by addition of TGF-β1 (1 ng/ml) or BMP-6 (100 ng/ml) for 30 min and were then immunoblotted using the indicated antibodies. Tubulin served as a loading control. Blots were cropped and the full-length blots are presented in Supplementary Figure S5. Bands intensities of PEG10 and tubulin (lanes 1–3 and 8–10) were quantified using ImageJ software (**g**). The band intensity of PEG10 was normalised to that of tubulin.

*Peg10* is an imprinted gene acquired from a retrotransposon that plays a crucial role in placenta formation during pregnancy, and deletion of *Peg10* in mice causes early embryonic lethalty^39^. Therefore, no information is currently available regarding the possible roles of PEG10 in the formation or maintenance of cartilage. We evaluated the tissue distribution of *PEG10* gene expression in normal adult tissues of humans and mice, and found extremely high expression of *PEG10* in the placenta, while it was moderately expressed in bone and cartilage (Supplementary Fig. S3a, b). Next, we compared *PEG10* expression levels among cultured normal or tumour cell lines of human bone and cartilage. Mesenchymal stem cell line, UBE6T-15, and the most widely examined osteosarcoma cell lines (MG63, HOS, and Saos-2), as well as C28/I2 chondrocytes, showed suppressed *PEG10* expression compared with the normal human osteoblast cell line, hFOB 1.19, whereas chondrosarcoma cell lines, SW1353 and Hs 819.T, exhibited over 1.5-fold higher levels (Fig. 3a). These results suggested relatively specific expression of *PEG10* in cartilage tumour cells. Because the larger protein variant RF1/RF2 (~140 kDa) is reported to be synthesised by a retroviral -1 ribosomal frame shift in the *PEG10* mRNA at the C-terminal region of RF1^34,38^, in addition to the major PEG10 translation product (called RF1 protein, 50–55 kDa), we checked its expression by immunoblotting using an antibody capable of detecting both RF1 and RF/RF2 proteins. We detected a band that migrated between 63 and 48 kDa that we considered to be the PEG10-RF1 protein, which was specifically abolished by PEG10 siRNA (siPEG10) transfection (Fig. 3b). This protein band was indeed stronger in chondrosarcoma cell lines, SW1353 and Hs 819.T, than in C28/I2 chondrocytes. The strong band of 180 kDa detected in chondrosarcoma cells was not silenced by the siRNA, indicating that the band was a non-specific protein detected by the antibody and that the PEG10 RF1/RF2 protein was not expressed in chondrosarcoma cells and chondrocytes, because no other band around 140–180 kDa was affected by siPEG10. Next, to investigate the molecular mechanism underlying the mutually exclusive expression of PEG10 and TGF-β/BMP signalling in cartilage tumours, we determined whether exogenous application of TGF-β or BMP reduced the PEG10 level. In C28/I2 normal chondrocytes, TGF-β1 did not inhibit the level of *PEG10* but rather it enhanced it, while BMP-6 showed no effect (Fig. 4a). However, in Hs 819.T chondrosarcoma cells, application of TGF-β1 suppressed *PEG10* expression (Fig. 4b). Interestingly, BMP-6 heightened the *PEG10* level (Fig. 4b). Similarly, in SW1353 chondrosarcoma cells, expression of *PEG10* had declined 48 h after TGF-β1 induction in a dose-dependent fashion (Fig. 4c), reproducing the results of microarray analysis. In contrast, the *PEG10* expression level was dose-dependently increased by BMP-6 stimulation (Fig. 4c). To confirm this trend at the protein level and to determine whether TGF-β1 or BMP-6 directly affect PEG10 expression within 60 min of application, we performed immunoblotting before and 10, 30, 60 min, and 2 days after induction. A similar result as shown in Fig. 4c was obtained at 2 d, indicating different roles of exogenous TGF-β and BMP in the regulation of PEG10 expression, whereas the PEG10 protein level was unchanged within 60 min of ligand induction, suggesting that PEG10 is not a direct target of TGF-β or BMP signalling (Fig. 4d,e). However, this BMP-induced elevation of PEG10 expression in chondrosarcoma cells *in vitro* was inconsistent with our results from clinical specimens of enchondroma and chondrosarcoma; the activity of the BMP-SMAD pathway and expression of PEG10 were mutually exclusive (Figs 1 and 2). To investigate the contribution of endogenous TGF-β and BMP signalling to regulate PEG10 expression, we blocked each pathway by applying specific receptor inhibitors SB431542^40^ and LDN193189^41^, respectively. As positive controls, SB431542 and LDN193189 inhibitors completely abolished TGF-β1-induced SMAD3 phosphorylation and BMP-6-mediated activation of SMAD1/5/9, respectively, in both SW1353 and Hs 819.T chondrosarcoma cell lines (Fig. 4f, lanes 4–7 and 11–14). Treatment with SB431542 indeed mildly increased the basal level of PEG10 in both SW1353 and Hs 819.T cells, indicating the inhibitory role of endogenous TGF-β signalling against PEG10 expression (Fig. 4f,g). However, LDN193189 did not decrease the level of PEG10, suggesting that endogenous BMP signalling was dispensable for PEG10 induction (Fig. 4f,g).

### PEG10 mildly interferes with canonical TGF-β/BMP signalling

**Figure 5:**
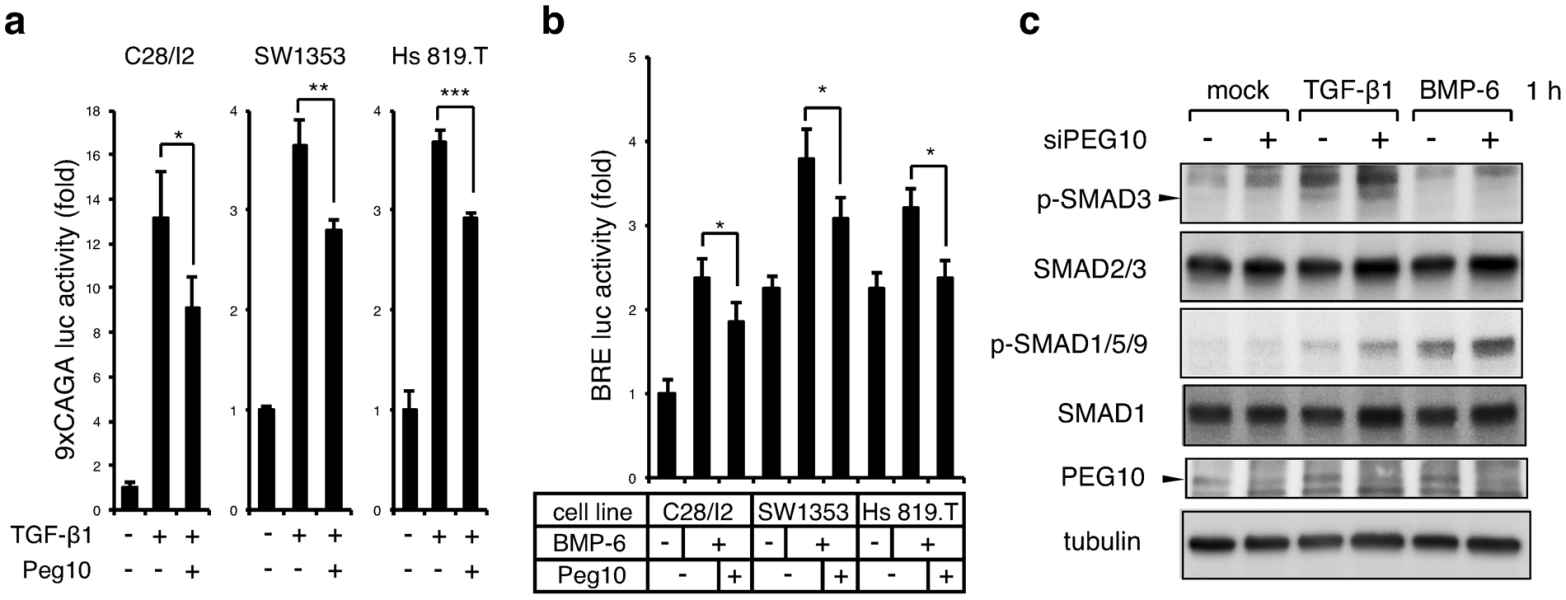
Canonical TGF-β/BMP signalling is mildly suppressed by PEG10. (**a**,**b**) The 9xCAGA or BRE luciferase reporter plasmid together with a *Renilla* reporter were transfected with or without a *Peg10* expression vector followed, 8 h later, by stimulation with TGF-β1 (1 ng/ml) or BMP-6 (100 ng/ml) overnight, respectively. Firefly reporter activity was normalised to Renilla activity (n = 3). *P < 0.05; **P < 0.01; ***P < 0.001. (**c**) SW1353 chondrosarcoma cells were transfected with control or PEG10 siRNAs followed, 12 h later, by stimulation for 1 h with TGF-β1 (1 ng/ml) or BMP-6 (100 ng/ml). Cells were then immunoblotted using the indicated antibodies. Tubulin served as a loading control. Blots were cropped and the full-length blots are presented in Supplementary Figure S6.

To determine whether PEG10 is capable of blocking activation of type I receptors of TGF-β/BMP signalling in chondrosarcoma cells, as previously shown in the RIB mink lung cell line^38^, we performed reporter luciferase assays. The V5-tagged mouse Peg10-RF1 expression plasmid was transfected into chondrosarcoma cells. Substantial transgene expression was confirmed with anti-V5 and anti-PEG10 antibodies by immunoblotting (Supplementary Fig. S4). Induction of Peg10 mildly but significantly inhibited the activity of TGF-β-SMAD2/3-responsive 9xCAGA luc in not only C28/I2 chondrocytes but also chondrosarcoma cell lines (Fig. 5a). A similar inhibitory action of Peg10 was observed in the BMP-SMAD1/5/9-specific BRE luc reporter assay (Fig. 5b). Conversely, 1 h after ligand stimulation, silencing of PEG10 moderately enhanced TGF-β1-induced activation of SMAD3 in SW1353 cells, and substantially increased BMP-6-mediated phosphorylation of SMAD1/5/9 (Fig. 5c). These results revealed mild but notable interference by PEG10 against both canonical TGF-β and BMP signalling in chondrosarcoma cells.

## Discussion

**Figure 6:**
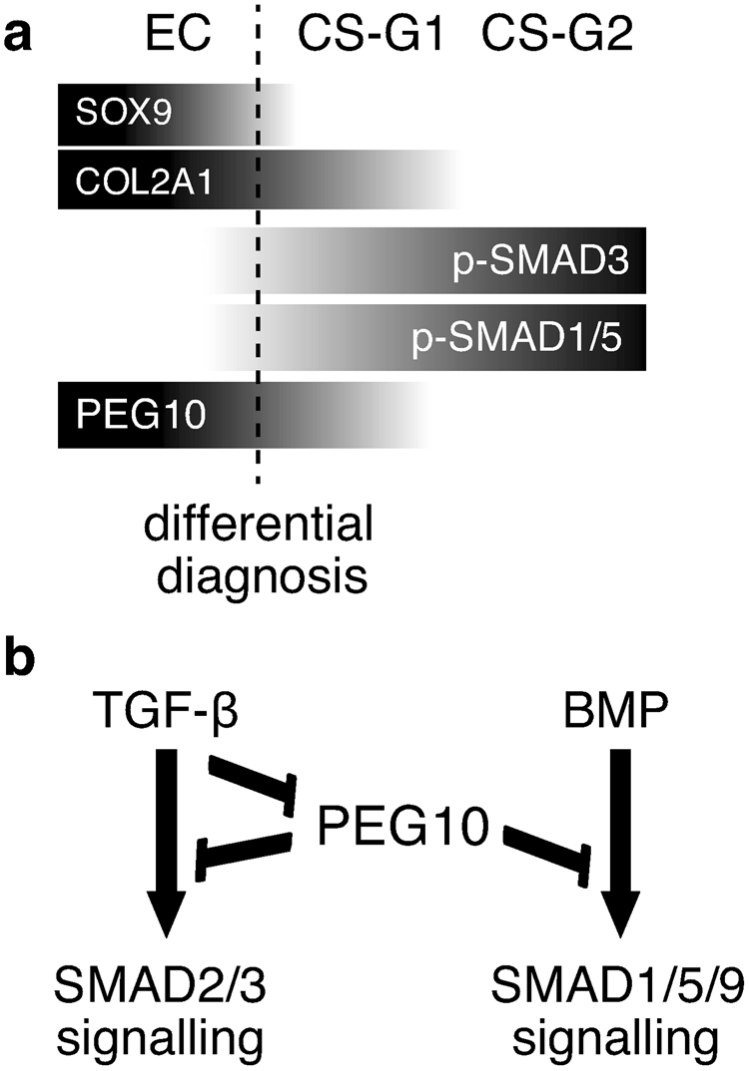
Diagrams illustrating the two major findings of this study. (**a**) Mutually exclusive expression patterns of PEG10 and TGF-β/BMP signalling molecules (p-SMADs), as well as differential expression of SOX9, which discriminate between enchondroma and chondrosarcoma (broken borderline), might be used for differential diagnosis of chondrosarcoma and enchondroma. COL2A1 might be a marker to distinguish grade 2 from grade 1 chondrosarcomas. (**b**) TGF-β signalling inhibits expression of PEG10, while PEG10 interferes with both SMAD pathways of TGF-β and BMP. Therefore, TGF-β signalling and PEG10 are mutually inhibitory in chondrosarcoma cells.

Our study has two major findings that are illustrated in Fig. 6. First, p-SMAD3 and p-SMAD1/5 accumulated in nuclei of chondrosarcoma cells, whereas PEG10 was abundantly expressed in enchondroma cells, indicating that expression of PEG10 and activated R-SMADs were mutually exclusive between these benign and malignant cartilage tumours (Fig. 6a). In addition, the chondrogenic master regulator, SOX9, showed differential expression between enchondroma and grade 1 chondrosarcoma, while expression of the cartilage-specific gene, *COL2A1*, was decreased in grade 2 chondrosarcoma compared with grade 1 (Fig. 6a). Second, PEG10 expression was suppressed by TGF-β1 stimulation in chondrosarcoma cells, while activation of the R-SMAD pathway by TGF-β1 or BMP-6 stimulation was mildly inhibited by PEG10 (Fig. 6b). Thus, the TGF-β-SMAD pathway and PEG10 are mutually exclusive and inhibitory in cartilage tumours.

Studies regarding the expression of TGF-β family signalling components in chondrosarcoma have reported controversial results. Masi *et al*. examined the expression of three TGF-β isoforms and TGF-β receptor type I/II in 24 chondrosarcomas (10 grade 1 and 14 grade 2/3), five enchondromas, and five osteochondromas by IHC and RT-PCR^27^. They found significantly higher expression of TGF-β1 and TGF-β2 in grade 2 and 3 chondrosarcomas compared with grade 1 tumours. Importantly, the overexpression of TGF-β1 in chondrosarcomas was significantly associated with shorter disease-free survival. Boeuf *et al*. analysed 10 grade 1, 11 grade 2, and six grade 3 chondrosarcomas for expression of TGF-β/BMP ligands and type I receptors by RT-PCR, and phosphorylation of SMAD1/5/9 and SMAD2 by IHC^28^. They found strong activation of both SMAD1/5/9 and SMAD2 in chondrosarcomas in a grade-dependent fashion, while a high rate of phosphorylated SMAD2 was associated with shorter metastasis-free survival. However, in contrast to the report by Masi *et al*., Boeuf *et al*. found that expression of neither TGF-β1 nor TGF-β2 was increased in chondrosarcomas compared with normal cartilage, while BMP-2 expression was dramatically decreased and BMP-7 expression was increased. In addition, type I BMP receptor, ALK2, was upregulated in grade 3 chondrosarcomas compared with type 1 tumours. Importantly, Masi *et al*. did not detect any difference in TGF-β expression between enchondromas and chondrosarcomas, whereas Boeuf *et al*. did not examine enchondroma samples in their study. Therefore, the possible difference of TGF-β/BMP signalling activity between enchondromas and low-grade chondrosarcomas remained unclear. We analysed seven enchondromas, and 11 grade 1 and seven grade 2 chondrosarcomas to evaluate the activity of SMAD3 and SMAD1/5. Our present study is the first to show differential activation of TGF-β R-SMAD (p-SMAD3) between enchondroma and low-grade chondrosarcoma. Our finding of increased TGF-β/BMP signalling in grade 2 chondrosarcomas compared with grade 1 tumours (Fig. 1) is compatible with two previous reports by Masi *et al*. and Boeuf *et al*. Moreover, our results agree with those of Boeuf *et al*., because BMP-2 was significantly downregulated in chondrosarcomas, while expression of TGF-β ligands and receptors were not increased (Supplementary Fig. S1a,b). We also found decreased expression of TGF-β/BMP-SMADs in chondrosarcomas (Supplementary Fig. S1e,g), whereas the phosphorylation of SMADs was increased (Fig., 1). Therefore, inhibitory molecules like PEG10 should be able to modify the signalling pathways. Taken together, our results confirm the trend shown in previous reports that TGF-β/BMP-SMAD signalling is accelerated in chondrosarcomas in a grade-dependent manner. We found that expression of the chondrogenic master regulator gene, SOX9, was strong in enchondromas and significantly diminished in grade 1 chondrosarcomas, while expression of the cartilage-specific collagen gene, *COL2A1*, was dramatically decreased in grade 2 chondrosarcomas (Fig. 1c,d,e). This is the first study to confirm that chondrosarcomas lose the cartilage differentiation program as they gain a malignant phenotype. Indeed, chondrosarcoma cell lines did not respond to TGF-β1 for chondrogenesis (Supplementary Fig. S2a). SOX9 might be used as a marker to distinguish grade 1 chondrosarcoma from enchondroma, while *COL2A1* may be able to discriminate between grade 1 and 2 chondrosarcomas. *COL2A1* is frequently mutated in chondrosarcoma, that the mutations were accumulated across the gene footprint of *COL2A1*, supporting the notion of a transcription-associated mutation^42^. Therefore, the down-regulation of *COL2A1* in our chondrosarcoma samples might be a result of gene mutation that is independent of a decrease in the upstream regulator, SOX9. A weakness of our study is the small sample numbers, as is the case for the reports of Masi *et al*. and Boeuf *et al*. In addition, we were unable to analyse grade 3 chondrosarcoma samples. Because chondrosarcoma is a relatively rare tumour, we need to increase the sample number by, for example, forming a consortium with multiple universities and laboratories to share clinical samples.

PEG10 is overexpressed in various cancer types including, prostate cancer^37^, B cell lymphoma^43,44^, lung cancer^36^, gallbladder adenocarcinoma^45^, and hepatocellular carcinoma^35,46–48^. Our study is the first to describe the expression profiles of PEG10 in a kind of sarcoma, a tumour of mesenchymal tissue origin. In carcinomas, expression of PEG10 was associated with increased cell growth, tumour progression, and poor prognoses. Indeed, we found increased PEG10 expression in chondrosarcoma cell lines and enchondroma specimens. However, our findings in chondrosarcoma samples conflict with results from other cancers; i.e. PEG10 was attenuated in malignant chondrosarcoma in a grade-dependent fashion (Fig. 2), suggesting that the expression pattern and roles of PEG10 in cartilage tumours are different from those in carcinomas. We showed that TGF-β1 diminished expression of PEG10 at day 2. PEG10 is a direct target of c-Myc, which is upregulated in hepatocellular carcinoma^46^, while c-Myc is a downstream target of the TGF-β-SMAD pathway, which is downregulated^49^. Hence, TGF-β may suppress PEG10 expression by inhibition of c-Myc expression. However, we found that neither knockdown nor forced expression of c-Myc affected PEG10 expression in SW1353 cells (data not shown). Indeed, Lux *et al*. reported that induction of exogenous c-Myc does not enhance PEG10 expression in HEK293 and HepG2 cells^50^. Future studies regarding the promotor region of the *PEG10* gene with TGF-β stimulation may reveal the transcription mechanism of *PEG10*. Lux *et al*., reported that PEG10-RF1 forms complexes with both type I and II receptors of TGF-β and BMP, and interferes with their activity *in vitro*, although the inhibitory effect is not drastic^38^. We obtained similar results in TGF-β and BMP signalling reporter luciferase assays with chondrosarcoma cells transfected with PEG10-RF1, which mildly inhibited both pathways (Fig. 5a,b). Because this moderate inhibitory action of PEG10 was confirmed at the endogenous level (Fig. 5c), the high expression of PEG10 in enchondroma should be responsible for the low rate of SMAD phosphorylation (Figs 1 and 2). Conversely, in chondrosarcomas, the low level of PEG10 expression appeared to be associated with high SMAD phosphorylation. Thus, our finding of the mutually exclusive expression patterns of PEG10 and p-SMADs between enchondroma and chondrosarcoma might be applied as a combined molecular marker to distinguish these cartilage tumours (Fig. 6a).

TGF-β signalling plays well-known dual roles in carcinogenesis. At early (low-grade) stages, it suppresses cell growth via induction of CDK inhibitors and downregulation of the cell cycle driver c-Myc. During late (high-grade) stages, TGF-β plays pro-oncogenic and pro-metastatic roles via promotion of EMT by inducing expression of Snail/Slug through the SMAD pathway^51,52^. Masi *et al*. showed that chondrosarcomas that overexpress TGF-β1 and TGF-β2 have significantly higher expression of the cell proliferation marker, MIB-1^27^, whereas Boeuf *et al*. found no effect of TGF-β or BMP signalling on the growth of chondrosarcoma cell lines, JJ012 and SW1353^28^. Therefore, the roles of TGF-β and BMP signalling in the growth of chondrosarcoma remain elusive. *In vitro*, TGF-β increases the motility of JJ012 chondrosarcoma cells^53–55^. In the case of enchondroma with high PEG10 expression, PEG10 suppresses TGF-β signalling and may prevent malignant tumour progression, for example the gain of cell motility. In future studies we need to examine possible roles of PEG10 in the growth and invasiveness of chondrosarcoma cells.

In conclusion, we demonstrated that expression of PEG10 and p-SMADs is mutually exclusive among enchondromas and chondrosarcomas. PEG10 is downregulated by TGF-β signalling, while PEG10 interferes with the TGF-β/BMP-SMAD pathway. A high ratio of p-SMAD3 (or p-SMAD2)/PEG10 may be a determinant for cartilage tumours to gain malignancy. In addition to PEG10, SOX9 was significantly attenuated in grade 1 chondrosarcoma compared with enchondroma. Thus, the combination of PEG10 and p-SMADs together with SOX9 might be used for differential diagnosis of chondrosarcoma and enchondroma.

## Methods

### Enchondroma/chondrosarcoma tissue cohort

Chemotherapy/radiotherapy naïve tumour specimens were collected from patients undergoing surgical resection or core biopsy at Kagoshima University between 2006 and 2015: seven enchondromas (three males and four females), 11 grade 1 chondrosarcomas (three males and eight females), and seven grade 2 chondrosarcomas (three males and four females). The specimens were processed into FFPE tissue blocks and/or frozen for subsequent RNA purification. One normal bone specimen for RNA analysis was obtained from a tumour-free bone region of an amputated leg (a male patient with grade 1 chondrosarcoma). Pathological diagnosis of tumours was performed by skilled pathologists in the Departments of Pathology and Orthopaedic Surgery of Kagoshima University. Written informed consent for examination of surgically excised tissue was obtained from patients. This study was approved by the Ethics Committee on Clinical Research at Kagoshima University Hospital (Protocol # 27–29). All methods were carried out in accordance with Ethical Guidelines for Medical and Health Research Involving Human Subjects.

### Cell lines and reagents

Chondrosarcoma cell lines, SW1353 and Hs 819.T, were obtained from the American Type Culture Collection (ATCC) and cultured in Dulbecco’s modified Eagle’s medium (DMEM)/Ham’s F-12 (1:1) (Invitrogen) supplemented with 10% foetal bovine serum (FBS). Human normal chondrocyte cell line, C28/I2, was a kind gift from Dr. Mary Goldring^56^. Micromass culture was performed by seeding cells on plates as 10 μl drops of cell suspension (1 × 10^6^ cells/ml) for 2 h to form 3-D cell masses that were subsequently covered with culture medium. Alcian blue and ALP staining were performed with alcian blue 8GX (Sigma) and an ALP staining kit (#85L-3R, Sigma), respectively. Human mesenchymal stem cell line, UBE6T-15, was obtained from the Japanese Collection of Research Bioresources (JCRB) Cell Bank, and human primary foetal osteoblast cell line, hFOB 1.19, human osteosarcoma cell lines, MG-63, HOS, 143B, and Saos-2, and HepG2 cells were purchased from the ATCC. Cells were cultured in DMEM (Sigma) with 10% FBS. To stimulate cells, 1 ng/ml TGF-β1 (PeproTech) or 100 ng/ml BMP-6 (PeproTech) was applied. For serum-free culture, medium was supplemented with insulin/transferrin/selenium (ITS) (Sigma). Inhibitor compound SB431542 (Sigma) was applied at 1 μM, whereas LDN193189 (Sigma) was added at 0.1 μM. Dimethyl sulfoxide (DMSO) was used for the vehicle control at 0.1%. All culture media contained 100 U/ml penicillin G and 100 µg/ml streptomycin.

### IHC and IF

FFPE tissue blocks were sectioned at 4 µm thickness. Antigens were retrieved by incubation in citrate buffer for 20 min at 95 °C. CAS-Block (Life Technologies) was used for blocking. Sections were incubated with anti-PEG10 (1:200, 4C10A7, LSBio), anti-p-SMAD1/5 (S463/S465; 1:200, Invitrogen), anti-p-SMAD3 (S423/425; 1:500, Rockland) or anti-SOX9 (1:100, H-90, sc-20095, Santa Cruz Biotechnology) antibodies, followed by incubation with secondary antibody Histofine Simplestain MAX-PO (MULTI) and a DAB solution (Nichirei Bioscience). Normal rabbit or mouse IgG was used as negative controls. Mayer’s haematoxylin solution was used for counterstaining. Images of 10 independent fields per section were captured under a BX53 microscope equipped with a DP21 digital camera (Olympus). Semi-quantitative scoring of target protein staining was performed as reported previously^28^. Briefly, the percentage of positively stained cells (0 = 0%, 1 = 1–24%, 2 = 25–49%, 3 = 50–74%, and 4 = 75–100% positive) and the staining intensity (0 = negative, 1 = weak, 2 = moderate, and 3 = strong intensity) were evaluated, and the average score of 10 fields per section was calculated. The summed score of the percentage and the intensity of positive staining were analysed statistically. For IF, anti-mouse Alexa Fluor 488 (1:300, A11001, Invitrogen) or anti-rabbit Alexa Fluor 568 (1:300, A11011, Invitrogen) were used as secondary antibodies to detect signals. Fluorescent images were captured with an AX80 microscope and DP70 digital camera (Olympus).

### RT-qPCR

Cells were lysed with TRIzol reagent (Invitrogen) to purify RNA, and 1 µg RNA was reverse transcribed into cDNA using a Verso cDNA Kit (Thermo Scientific). Human multiple tissue cDNA panels (Human MTC Panel I and II) were purchased from Clontech. A mouse tissue cDNA panel was generated as described previously^57^. Animal experiments were approved by the Institutional Animal Care and Use Committee of Kagoshima University (# MD12137) and performed in accordance with Guidelines for Proper Conduct of Animal Experiments. The relative expression of gene transcripts was determined by qPCR using SYBR premix Ex Taq II (Takara) and a Thermal Cycler Dice TP850 (Takara). PCRs were performed in duplicate per sample, and the measured expression level of each gene was normalised to that of *GAPDH*. Experiments were performed in triplicate unless mentioned otherwise. Sequence information of primers is listed in Supplementary Table S1.

### Microarray analysis

C28/I2, SW1353, and Hs 819.T cells were treated with or without TGF-β1 (1 ng/ml) for 48 h. mRNA samples purified by TRIzol reagent were cleaned up using an RNeasy MinElute Cleanup Kit (Qiagen) and analysed on a human Gene 2.0 ST Array (Affymetrix). We screened for genes that had more than 4-fold higher expression levels in both SW1353 and Hs 819.T cells compared with C28/I2 cells with notable probe signal levels of >400, but that had decreased to less than 0.5-fold upon TGF-β1 stimulation in chondrosarcoma cells.

### siRNA-mediated knockdown of PEG10

Dharmacon ON-TARGETplus SMARTpool for *PEG10* (#L-032579-01; a mixture of four independent siRNAs against human *PEG10*) and the negative control non-targeting siRNA pool (#D-001810-10) were purchased from GE Healthcare. siRNAs were transfected into cells using Lipofectamine RNAiMax (Invitrogen).

### PEG10-expressing plasmid

Mouse *Peg10* cDNA was cloned from mRNA purified from mouse placenta by RT-PCR, subcloned into the entry vector, pENTR, and then cloned into the C-terminally V5-tagged expression vector, pEF-DEST51 (Invitrogen). Experiments using plasmids were approved by the Kagoshima University safety control committee for gene-recombination techniques (# 27020). All experiments were performed in accordance with the Act on the Conservation and Sustainable Use of Biological Diversity through Regulations on the Use of Living Modified Organisms (Type 2 Use of Living Modified Organisms).

### Immunoblotting

For immunoblotting, cells were lysed in M-PER lysis buffer (Thermo Scientific) containing aprotinin, sodium orthovanadate, and phenylmethylsulfonyl fluoride, and then subjected to SDS-polyacrylamide gel electrophoresis, protein transfer, and chemiluminescence using standard protocols. Blots were incubated with anti-PEG10 (1:1,000, 4C10A7, LSBio), anti-Smad1 (1:1,000, # 9743, CST), anti-p-Smad1/5/9 (1:1,000, D5B10, CST), anti-Smad2/3 (1:1,000, # 610842, BD Biosciences), anti-p-Smad3 (1:1,000, C25A9, CST), or anti-tubulin (1:1000, DM1A, Sigma) antibodies and then with horseradish peroxidase-conjugated anti-rabbit or anti-mouse secondary antibodies (1:10,000, CST). Chemiluminescent signals were detected using an LAS 4000 Mini Image Analyzer (Fujifilm). Band intensities of PEG10 and tubulin were quantified using ImageJ 1.50i software (National Institutes of Health, USA). The band intensity of PEG10 was normalised to that of tubulin.

### Luciferase assay

Cells were seeded in triplicate in 24-well plates and transfected with 9xCAGA or the BRE luciferase reporter plasmid (a kind gift from Dr. Kohei Miyazono, the University of Tokyo) and the pGL4.75hRlucCMV Renilla vector (Promega) with or without the expression vector for Peg10. Dual luciferase assays were performed as described previously^58^ using a GloMax 96 microplate luminometer (Promega).

### Statistics

Results are expressed as the mean ± standard deviation of at least three independent experiments. Statistical comparisons between the various treatments were performed using the unpaired Student *t*-test. A value of *P* < 0.05 was considered significant.

## Electronic supplementary material

Supplementary Files
