# Supplementary material for: TGF-β signalling and PEG10 are mutually exclusive and inhibitory in chondrosarcoma cells
Source: Sci Rep. 2017 Oct 18;7:13494. doi: 10.1038/s41598-017-13994-w (PMC5647403; doi:10.1038/s41598-017-13994-w)

Supplementary Information

TGF-β signalling and PEG10 are mutually exclusive and inhibitory in chondrosarcoma cells

Naohiro Shinohara[1,2†], Shingo Maeda[1†*], Yuhei Yahiro[1,2], Daisuke Sakuma[1,2], Kanehiro Matsuyama[2], Katsuyuki Imamura[2], Ichiro Kawamura[2], Takao Setoguchi[3], Yasuhiro Ishidou[1], Satoshi Nagano[2], & Setsuro Komiya[1,2,3]

[1]Department of Medical Joint Materials, Kagoshima University, Kagoshima, Japan

[2]Department of Orthopaedic Surgery, Kagoshima University, Kagoshima, Japan

[3]The Near-Future Locomotor Organ Medicine Creation Course, Kagoshima University, Kagoshima, Japan

[†] These authors contributed equally to this work.

Supplementary Table S1

Primer sequences utilized in this study (5' to 3')

Human

Gene	Forward	Reverse
SOX9	AGTACCCGCACTTGCACAAC	TTCAGAAGTCTCCAGAGCTTGC
COL2A1	TCTGGGTTCCAGGGACTTCCT	GAACACCCTGGTCACCTGGTTTT
PEG10	CGAAGAAGGGACGAGCTCTCTG	CCTCCGACTGCTTCATGACCTT
TGFB1	AATTGAGGGCTTTCGCCTTAGC	AACCCTGCGTTGATGTCCACTT
TGFB2	CAAAATAGACATGCCGCCCTTC	CCATTGCTGAGACGTCAAATCG
TGFB3	AGCACAACGAACTGGCTGTCTG	GGACACATTGAAGCGGAAAACC
BMP2	CATGATGGAAAAGGGCATCCTC	TTAAGGCGTTTCCGCTGTTTGT
BMP6	TGGAGAATGCTCCTTCCCACTC	CGGGGTTCATAAGGTGAACCAA
GDF2 (BMP-9)	GGTTCCAGAAACCTGCCCTTCT	ATGGCTGATCATCTCCCTCAGC
TGFBR1 (ALK5)	TCCAAACCACAGAGTGGGAACA	TCGTCGAGCAATTTCCCAGAAT
ACVR1 (ALK2)	GGGCCTTTGGACTTGTTTTGTG	CACATCGTAGAACGGTGGCTTG
ACVR1B (ALK4)	GAGGCATCTGCATGGGTCTTCT	CCCTCCACCCTGATCAGACAGT
ACVR1C (ALK7)	GGCGCTCTCAATTGCTAGTGGT	ACCCTAAGTCCGCTATGGCACA
BMPR1A (ALK3)	ATGCGTGAGGTTGTGTGTGTCA	GGGCCCAGCATTCTGACATTAG
BMPR1B (ALK6)	GTGCCCAGTGACCCCTCTTATG	GGGGCGTAACTTCTTGATGCAC
SMAD1	AGAGGAGATGTTCAGGCGGTTG	ACTTGTGGAGGAGGCATGGAAC
SMAD2	TGAAAGGGTGGGGAGCAGAATA	ATGCTTGAGCAACGCACTGAAG
SMAD3	CATGACTGTGGATGGCTTCACC	GCTGCATTCCTGTTGACATTGG
SMAD4	TGGGTCAACTCTCCAATGTCCA	TCCAACTGCACACCTTTGCCTA
SMAD5	ACCTGAGCCACAATGAACCACA	GAAGGGGGATAAGGGCTGTTTG
SMAD9	GCAGAGCCGGAACTGCAACTAT	AGCTGAGCGAAGAGCTGGTTGT
GAPDH	CACCATCTTCCAGGAGCGAGAT	TGGACTCCACGACGTACTCAGC

Mouse

Gene	Forward	Reverse
Peg10	AGCACGCATGACCCTCACATTA	TACGGATGCAAATTCGGTTGTG
Hprt1	CGTTGGGCTTACCTCACTGCTT	CAAAAAGCGGTCTGAGGAGGAA

Supplementary Figure S1

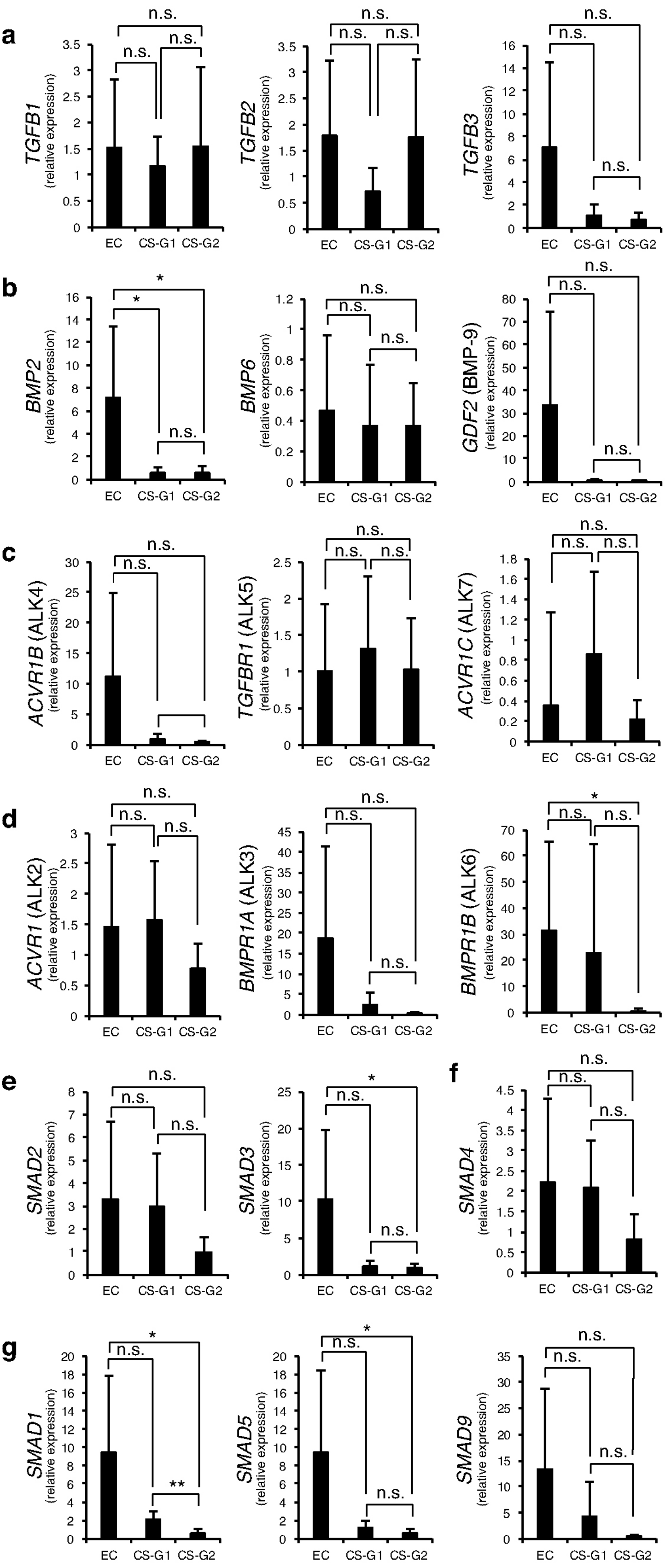

Supplementary Figure S1. Expression of genes encoding signalling components of TGF-β and BMP pathways. mRNA samples from tumours were examined by RT-qPCR. EC, enchondromas (n = 6); CS-G1, grade 1 chondrosarcomas (n = 5); CS-G2, grade 2 chondrosarcomas (n = 6). *$P < 0.05$; **$P < 0.01$; n.s., not significant.

Supplementary Figure S2

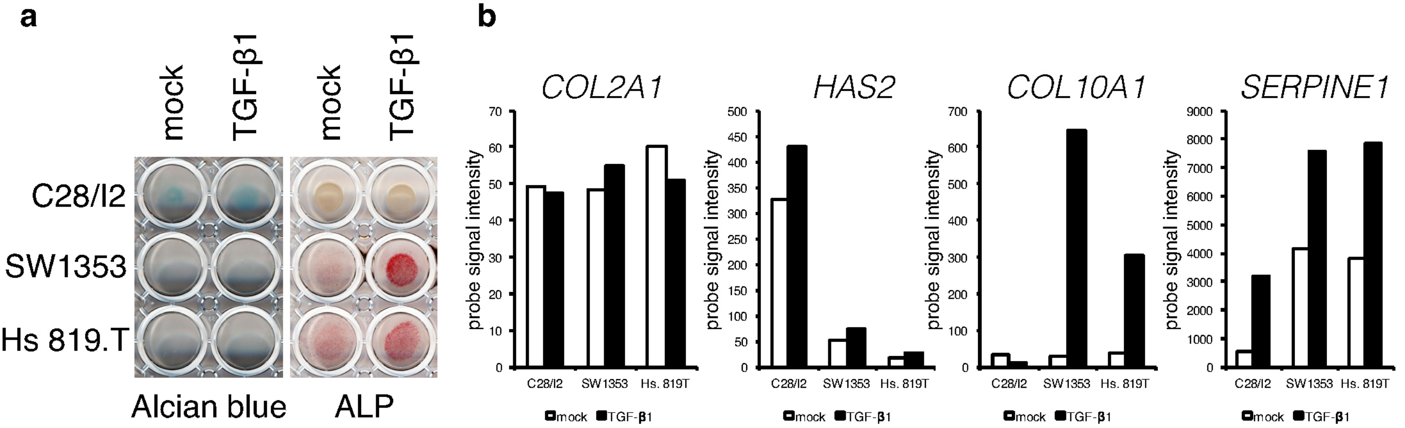

Supplementary Figure S2. Abnormal differentiation of chondrosarcoma cells upon chondrogenic stimulation by TGF-β1. (a) Micromasses of C28/I2, SW1353, and Hs 819.T cells were formed and then cultured further with or without TGF-β1 (1 ng/ml) for 7 days, followed by alcian blue and ALP staining. (b) Microarray analysis of *COL2A1*, *HAS2*, *COL10A1*, and *SERPINE1* expression in the experiment shown in Table 1.

Supplementary Figure S3

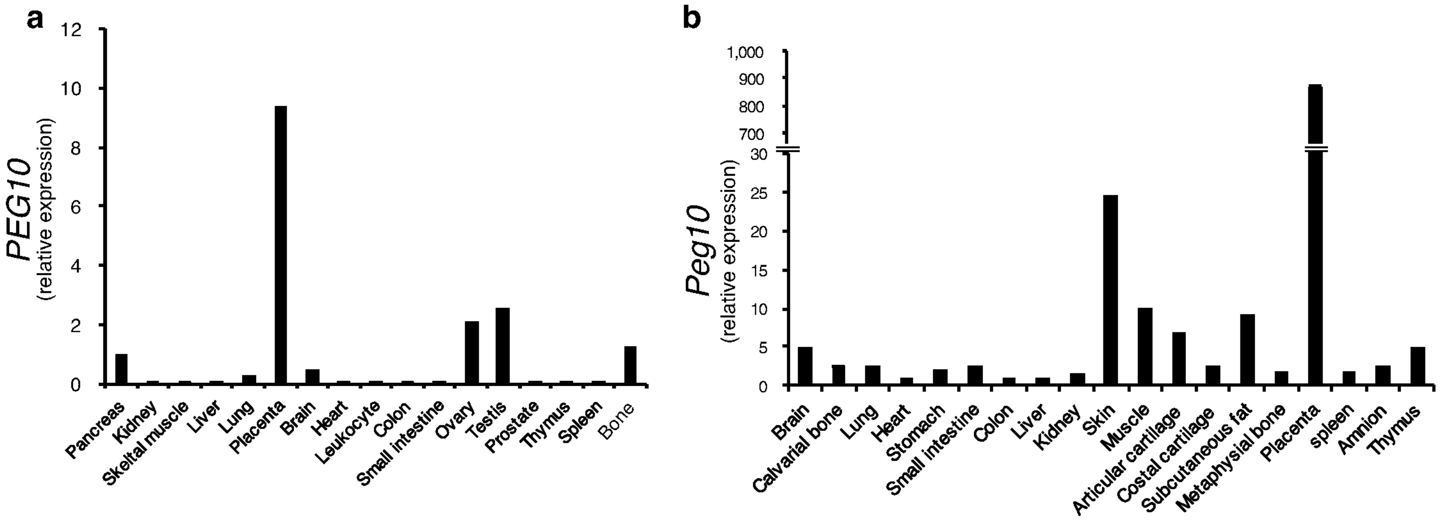

Supplementary Figure S3. Tissue distribution of PEG10 gene expression in humans and mice. (a, b) A multiple tissue cDNA panel of humans (a) and mice (b) was subjected to RT-qPCR analyses of *PEG10* (human) and *Peg10* (mouse).

Supplementary Figure S4

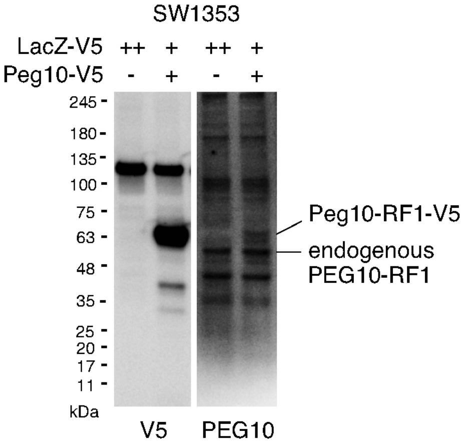

Supplementary Figure S4. Induction of exogenous PEG10 in SW1353 cells.

SW1353 cells were transfected with control LacZ or Peg10-V5 expression plasmids overnight, followed by immunoblotting with anti-V5 or anti-PEG10 antibodies.

Supplementary Figure S5

Full-length blots of immunoblots illustrated in Figures 3 and 4.

Fig.3b

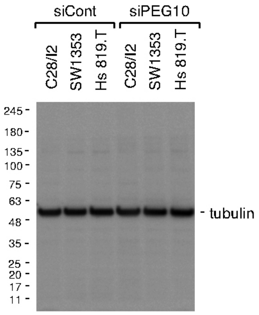

Fig.4d

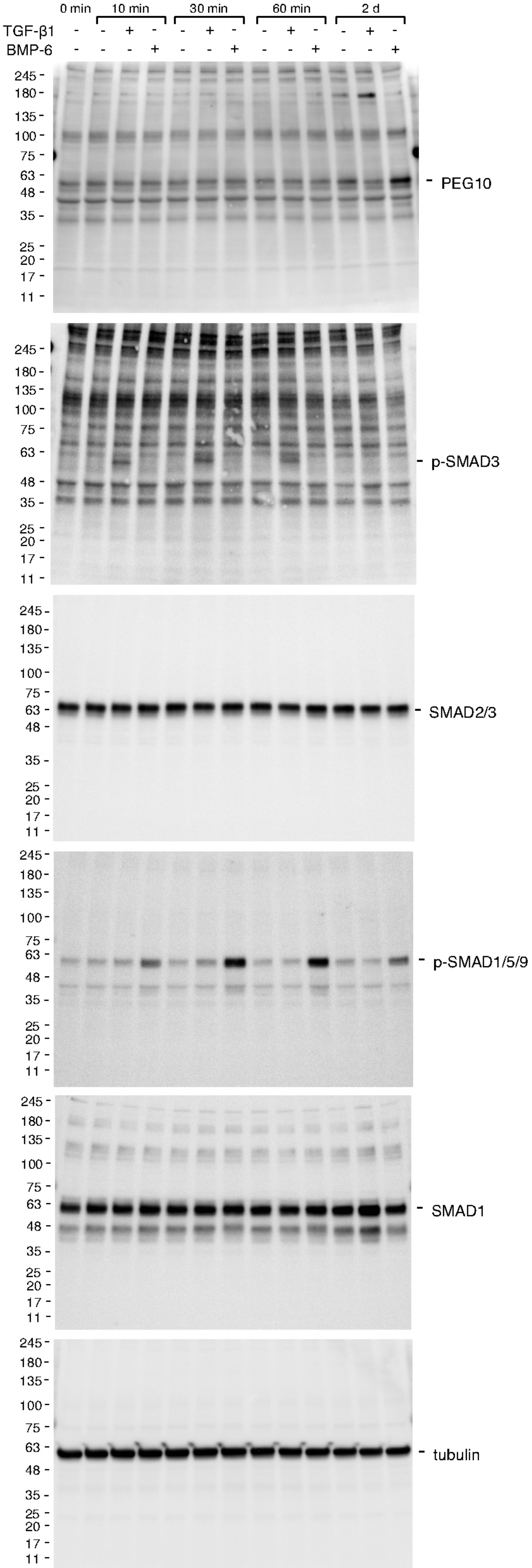

Fig.4f

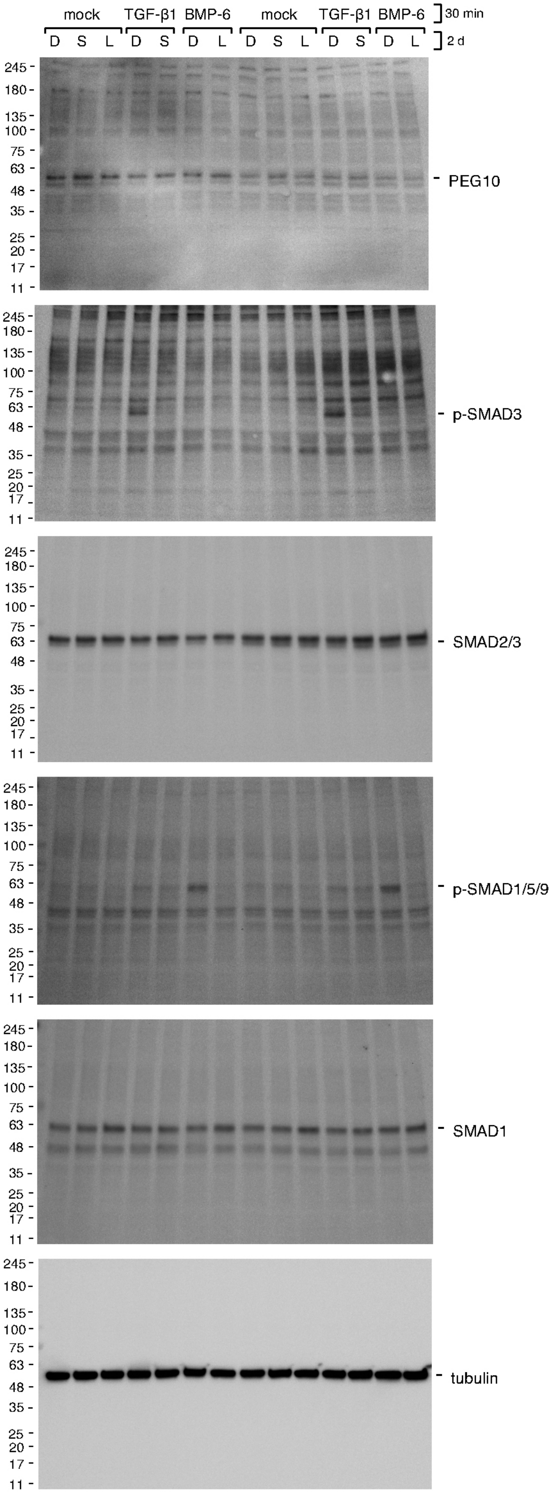

Supplementary Figure S6

Full-length blots of immunoblots illustrated in Figure 5c

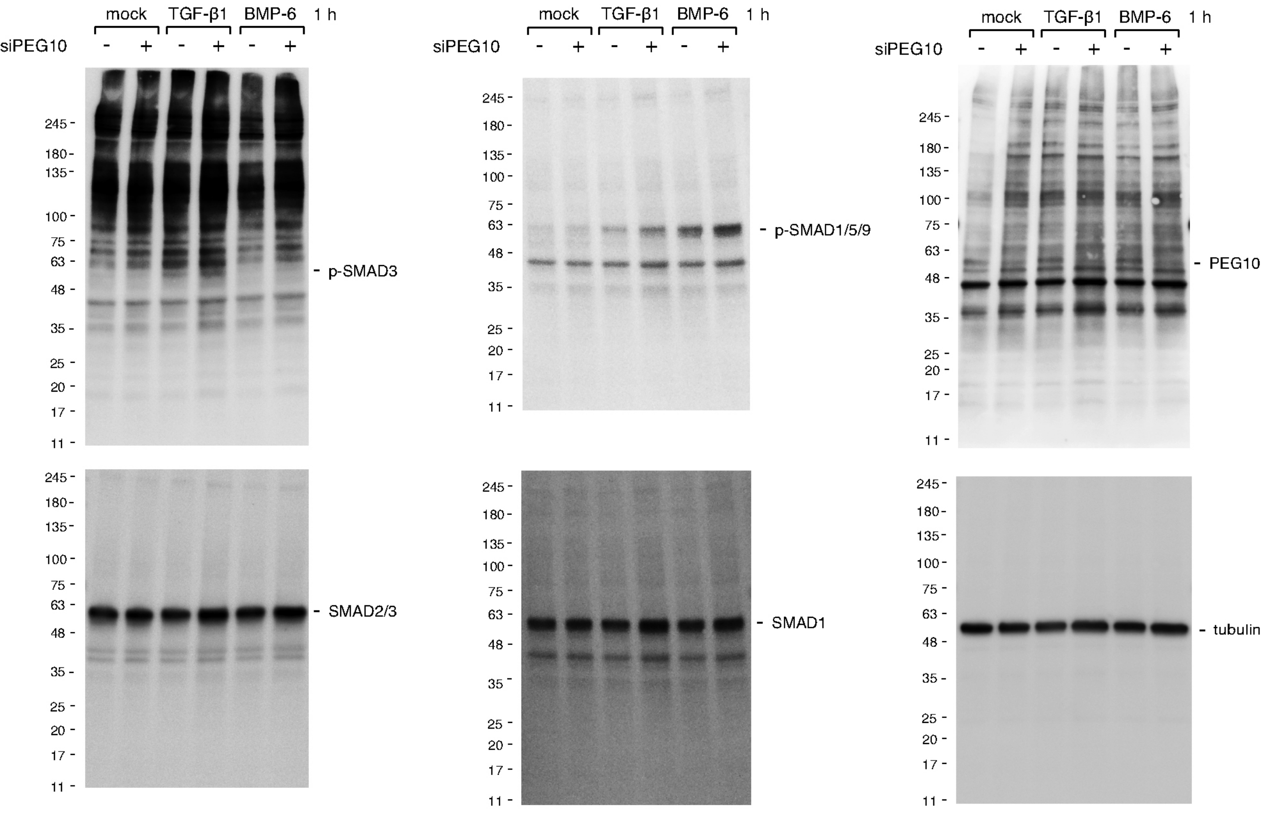

Supplement: Supplementary file 1 — Supplementary Files [file 41598_2017_13994_MOESM1_ESM.pdf]
